# The social determinants of health, law, and urban development: using human rights to address structural health inequalities in our cities

**DOI:** 10.1093/medlaw/fwac047

**Published:** 2022-11-18

**Authors:** Lisa Montel

**Affiliations:** Centre for Health, Law, and Society, University of Bristol Law School, UK; Cancer Survival Group, London School of Hygiene and Tropical Medicine, UK

**Keywords:** Health inequalities, Human rights, Right to health, Social determinants of health, Urban development, Urban planning, Legal determinants of health

## Abstract

The COVID-19 pandemic exacerbated profound inequalities in the conditions in which people live, work, and age. Law plays a critical role in shaping these structural health inequalities, which have existed for decades. This dynamic can be observed at the local level, with cities operating as environments unequally distributing the risks of non-communicable diseases between population groups. This article first focuses on urban development to explore the conceptual links between health inequalities and the role of law. I expand this observation and I posit that the social determinants of health are about human rights. With that in mind, I argue that human rights are necessary to address the issue of unequally unhealthy urban environments, hence recognising that people are entitled to a minimum essential level of the conditions in which they live, work, and age, which the State is responsible to fulfil. By way of strengthening my argument, I lay out how a human rights framework can improve these conditions and ameliorate unfair inequalities. Finally, I recognise and respond to the limits of a human rights approach.

## I. INTRODUCTION

In 2019, 83% of the population of England lived in urban areas.^1^ This proportion is expected to grow, with a faster growth rate in urban than in rural areas.^2^ Infectious diseases spread quickly in cities—as the recent COVID-19 pandemic showed—but ‘urbanisation is reshaping population health problems, particularly among poor people in urban areas, towards non-communicable diseases (NCDs), accidental and violent injuries, and effects of ecological disaster’.^3^ The organisation and design of urban spaces, such as isolated and dark streets, poor traffic management, lack of green spaces, high-density urban areas, or city sanitation practices, have an impact on daily conditions that shape our health and contribute to health inequalities. However, in practice, legal frameworks for decision-making in local government may pay little attention to health outcomes or may prioritise other considerations that may appear, *prima facie*, contradictory—such as promoting a strong economy. This raises a series of important, connected questions. How can the decision-making process be improved, in England, for Local Planning Authorities to promote health and reduce health inequalities? What is the role of the law in this? Can human rights contribute?

In this article, I explore the conceptual and practical links between urban development, health, law, and human rights. I bring together literature from global health, public health ethics, and human rights that seek to contribute to the shared goal of promoting human well-being. First, in Section II, I focus on the conceptual links, and I explain how research on the social determinants of health reveals the critical importance of the law in urban development and health. Then I discuss how law, conceptualised in a broad sense, may have an effect on the social determinants of health. In particular, I explore the law as a mechanism to shape structural power in our society, as a tool to regulate multiple areas interacting with urban development, and as a form of implementation of governance of public and private actors in urban planning in England. I argue that human rights cannot be discounted when researching the social determinants of health.^4^ In Section III, I make innovative use of global health scholarship to explore domestic questions on the application of legal mechanisms and tools to promote health and reduce health inequalities in urban development in England. I suggest applying a human rights framework, as defined in international human rights law, and thus partially implemented and enforceable through the UK’s domestic legal system. Finally, I conclude by reflecting on the added value and limits of this approach.

## II. URBAN DEVELOPMENT, LAW AS A STRUCTURAL DETERMINANT, AND HUMAN RIGHTS

### A. The social determinants of health in the context of urban development

Research on the social determinants of health shows how the daily conditions in which we are born, grow, live, work, and age, impact our health.^5^ These daily conditions are themselves determined by the unequal distribution of power, resources, and money in our society.^6^ The law, conceived in its basic meaning as ‘a rule […] [or set of rules] that is used to order the way in which a society behaves’, plays a key role in organising the distribution of power and resources in a society, and, therefore, as shaping the social determinants of health.^7^ In this section, I briefly introduce the social determinants of health and their conceptual links with the law, before applying this thinking to the context of urban environments in England.

#### 1. The social determinants of health and the law

By way of background, I use a definition of health that extends beyond the biomedical model—the absence of ill health—to encompass positive states of health and well-being.^8^ In terms of practice, policy, and law, I am interested in the creation or perpetuation of the social determinants of health.^9^ The findings from the World Health Organisation Commission on the Social Determinants of Health (WHO Commission), as well as the review of these findings 10 years later, show that the conditions of life, such as the level and quality of education, the availability of safe sanitation and potable water, the quality of the air one breathes, and the conditions of work, have a cumulative impact on the distribution of ill health across groups in the population.^10^ The WHO Commission observed a ‘social gradient in health’, meaning that health varies at each level of income, with worse health for each lower socio-economic level.^11^ These conditions of daily life are themselves impacted by the unequal distribution of power, money, and resources in our society.^12^ As a result, any research aiming at informing policies to reduce health inequalities and promote justice in public health should look beyond healthcare policy and view health as a product of our social system.^13^

Law plays a key role in the social determinants of health because it has a significant effect on the organisation and administration of our social system. In this article, I explore the role of law to improve the causes of the unequal distribution of ill health in the context of urban development. I am interested in health inequalities and NCDs; that is, diseases that are not transmissible and last for life, or a significant period in life. In the subsections below, I first posit that urban environments constitute an opportunity to observe how the law interacts with the social determinants of health. Then, I show how urban environments are, themselves, a social determinant of health.

#### 2. Observing the effect of law on the social determinants of health

Researching the causes of ill health at population levels is challenging on several counts, one of them being the multiplicity of variables—economic, political, social, biomedical, and behavioural—that interact with each other. For instance, we may imagine that an individual’s chances of getting lung cancer would depend on individual behaviour, such as smoking and alcohol use, genetic predispositions to developing this disease, and individual exposure to harmful agents, such as asbestos. Regular exposure to asbestos may be due to poor working conditions, lack of information on the dangers of exposure to such an agent, or lack of choice regarding one’s source of remuneration. Previous lung infections also have a cumulative effect on the chances of that individual to develop, one day, lung cancer. These previous infections may or may not have been treated fully depending on the availability and quality of health care received, which itself could depend on an individual's financial resources to enrol into a private health insurance scheme, and the quality of hospital care of the geographical area that individual lives in. Finally, additional exposure to air pollution may contribute to the development of cancerous cells in the lung, and to accelerating the spread of the disease.

In order to understand what laws, taken in a broad sense—primary and secondary legislation, jurisprudence, as well as the implementation of these rules (including through policy)—have an impact on health outcomes, it is necessary to take into account the time between the moment a law, a regulation, or a policy or programme is implemented, and the visible effect, if any, it has on health outcomes.^14^ For instance, experts estimate that a national cancer plan may take 8–10 years before its effect may be observable on national estimates of cancer survival.^15^

In the context of NCDs, there is an additional layer of complexity: the period of time between exposure to a harmful agent (air pollution or asbestos, for example) and the outcome (say, lung cancer). Indeed, NCDs are characterised by a plurality of causal factors, as well as the long time needed—sometimes years—for the first symptoms to manifest. This contrasts with infectious diseases, for which the incubation period is usually much shorter (for example, a few days), exposure to the virus can be located with more precision, and determining the cause(s) of a disease can thus be more accurate. With NCDs, the multiplicity of causal—or rather, contributing—factors, coupled with the lengthy period of time between exposure to a given factor and manifestation of the first symptoms, mean that social environments as a whole must be considered in order to understand and implement effective prevention.

In 2019 in England, 56.3 million people lived in urban areas (82.9% of the population).^16^ These areas are managed by 333 local authorities with devolved powers and funding to make decisions on the social determinants of health, such as housing, work, local economy, and health policies.^17^ In particular, the organisation and management of housing and neighbourhoods play a critical role in distributing health (in)equity among population groups.^18^ As such, urban environments represent a valuable opportunity to observe the interaction of political, economic, social, and cultural determinants of NCDs and their unequal distribution in the population. Urban environments represent what Venkatapuram calls ‘social environments’ in social epidemiology; that is, ‘economic, political, and social policies and processes driven by social and cultural values that create and distribute the daily conditions of life’.^19^

#### 3. Urban planning (law) as a social determinant of health

In addition to using urban environments as a laboratory, it is important to recognise that urban planning itself is a social determinant of health: ‘The planning and design of urban environments has a major effect on health equity through its influence on behaviour and safety’.^20^ The organisation, use, and management of land in urban areas have an effect on a myriad of health determinants that contribute to causing NCDs and deepening health inequalities. The WHO estimates that lack of physical activity, alcohol use, tobacco use, and unhealthy diets are the main causes of the four NCDs responsible for over 80% of premature NCDs deaths worldwide: diabetes, cancer, heart diseases, and respiratory diseases.^21^ Urban planning determines the quantity and quality of green space, the type and frequency of transport, including the design of cycle lanes, the quality and quantity of housing, including social housing, and the frequency and location of fast-food and off-licence shops. These conditions have a knock-on effect on the causes of NCDs, regarding, for example, physical activity, car use and cycling, air quality, sanitation, and indoor air quality, as well as dampness.

Planning law plays a critical role in this context. Planning law is the body of rules relating to the development; that is, ‘any building, mining, engineering or other operations which are carried out in, on, over or under land, or the making of material change of use of buildings or other land’.^22^ With that in mind, it is expected that planning law influences the social determinants of health. However, it is not the only field of law that does so. Urban development is concerned with a multitude of law fields, each having an effect on the social determinants of health. Before delving into these considerations, I will first explain how the law plays a role in shaping the unequal distribution of money, power, and resources. This is what the WHO Commission calls the ‘causes of causes’.^23^

#### 4. Inequities of power, money, and resources in urban development

The social determinants of health are themselves determined by structural issues of power, money, and resources.^24^ In England, the way planning works creates a ‘system of systems’ which is the result of a multitude of stakeholders having political and/or financial power to shape urban development.^25^ The power conferred to Local Planning Authorities is in tension with the informal power of private parties, such as developers. This is because planning development operates in a ‘plan-led’ system, which means that granting or refusing planning applications is decided on a case-by-case basis, depending on whether they fit with a given local development plan unless material considerations indicate otherwise.^26^ In essence, if a proposal ‘fits’ with policy criteria, the relevant planning application should be granted. The issue thus becomes a question of how well health is embedded into planning policy, and to what extent decision makers have discretionary power to interpret planning policy to promote well-being. Other priorities may trump health promotion, such as contributing to a strong economy.^27^ This approach to planning differs from a zonal system, where local authorities decide in advance what use different areas of land should be allocated; for example, residential or commercial. In this latter planning system, developers have more certainty, but Local Planning Authorities lose flexibility in deciding what is built on a particular piece of land. The proposed changes from the 2020 White Paper to planning law in England would have moved the system towards more zoning, but several parties voiced their concerns to this approach in a consultation.^28^ Until such changes are enacted, the planning system in England remains ‘plan-led’.

The (unequal) distribution of power, therefore, affects planning decisions which, in turn, have an effect on the determinants of NCDs and health inequalities. Researching the root causes of unhealthy urban development means looking at the role of law in shaping the structure of the social system, setting the rules within which urban development operates, as well as regulating powerful actors. I explore these three aspects below.

### B. Law as a structural determinant

A primary function of law is to regulate, -including by providing and limiting- power and setting the rules for an orderly, cohesive society. Law is the tool that materialises the social contract according to which citizens agree to give up a proportion of their autonomy to the State so that, in return, the State can regulate social, economic, and political interactions between its citizens.^29^ Law, and within it multiple areas of law, determine structural societal rules that shape the distribution of power, money, and resources. It also sets the rules for national policies, such as the National Planning Policy Framework (NPPF), the core policy informing all local development plans and decisions from Local Planning Authorities in England. A spectrum of influence of the law can thus be seen, ranging from the overarching structure that implements the political ideology in which we live— a capitalist market economy—through to the norms that regulate a plurality of areas and having an impact on urban health, including the daily rules that organise decision-making in urban development.

At one end of the spectrum, regulation (or lack thereof) pertaining to the market economy sets the background within which urban planning operates. In England, reliance on private actors to fund and deliver a public function, such as housing provision or road management, means that planning outcomes are influenced by the way these private actors operate. For instance, real estate investors are looking for high and/or regular financial returns, which means that they invest in projects with a potential to generate income. In this context, social projects, or projects that prioritise new outcomes, such as social equality and health, may represent financial uncertainty, which arguably is a risk that investors are less likely to take. In turn, developers are dependent on investors to fund the design and construction of new projects. In this context, Local Planning Authorities appear to have limited power over what gets built and how; a power which may be even more limited in the ‘plan-led’ English urban planning system.^30^

Beyond the law as implementing the politico-economic ideology in which society, and within it, planning operates, the law regulates a multiplicity of fields having an impact on urban development and health. Indeed, several areas of law operating in, and in parallel to planning, have an impact on urban development, and, in turn, on the prevention, protection, and promotion of health in urban areas.^31^ By way of illustration, the recent inclusion in England of a 10% social value in procurement rules contributes to determining the quality of large development schemes.^32^ Similarly, building regulations are known to have a significant impact on health, as the 2017 fire at the Grenfell Tower tragically testifies.^33^ Other areas include administrative law and its impact on the good governance of Local Planning Authorities, highways regulations and their impact on traffic, laws governing taxation, uses of buildings, and so on.^34^ All these areas of law influence urban planning—what gets built, where, and how—and have a significant impact on shaping the environment we live in, with the corresponding consequences on our health.

By way of illustrating the variety of law fields that impact health in urban development, I will now discuss some concrete policy examples which have had direct and indirect effects on health.

#### 1. The direct effect of laws on health in urban development

The NPPF pays particular attention to environmental concerns by listing sustainable development as the objective of urban planning in England.^35^ In this context, environmental law influences public and private actors’ decisions by imposing standards on the built environment. For instance, the ‘net zero’ policy in the UK, which aims to transition cities and regions to zero carbon emissions by 2050, is expected to have an impact on air quality, which in turn has an effect on respiratory diseases, some cancers, cardiovascular diseases, and diabetes.^36^ In 2019, the WHO published a report suggesting that air quality is the second leading cause of death from NCDs.^37^ A national policy aiming at reducing greenhouse gas emissions, therefore, has a direct impact on the prevention of NCDs (if successfully implemented).

Another example of the direct impact of laws on health is within the planning law itself; for example, a national policy aiming to increase access to green spaces for all communities in urban areas. Public Health England, an executive agency of the Department of Health and Social Care that has now been replaced by the Office for Health Improvement and Disparities and the UK Health Security Agency, collected evidence showing how access to green spaces has an impact on physical health, notably levels of cholesterols, cortisol, variability of heart rate, and blood pressure; as well as mental health outcomes such as depression, levels of anxiety and stress, as well as resilience,^38^ which is in line with WHO findings and policy recommendations.^39^ Evidence also suggests that access to green spaces benefits more deprived communities and may, therefore, help in reducing health inequalities.^40^ Finally, transport and, in particular, the promotion of cycle lanes provide another example of the direct impact of laws on health in urban development. Evidence suggests that walking and cycling infrastructure in urban development is associated with increased walking and cycling, which, in turn, increases physical activity.^41^

These examples show how laws may have a direct impact on health in urban planning. These laws are by no means limited to those studied within the siloed fields of healthcare law or planning law. They extend to fields beyond health such as environmental law, administrative law, and transport. In addition, laws may have an indirect effect on health outcomes in urban development.

#### 2. The indirect effect of laws on health in urban development

Laws may indirectly impact health in two ways: by regulating outcomes and by regulating behaviours. By way of example, consider a national policy aiming at increasing the number of housing accommodations; for instance, the UK’s target to achieve 300,000 new homes a year.^42^ Such a policy may have indirect positive and negative effects on health. If the policy leads to an emphasis on the number of new homes over the quality of housing and affordability, it may result in decisions favouring expensive and/or poorly built houses. Standards necessary to promote good physical and mental health may not be respected, such as the Decent Home standards in England or the WHO housing standards having an impact on health.^43^ A national policy emphasising housing targets may also overlook the accessibility of housing to persons with physical disabilities and older people.^44^

In addition to the indirect impact of laws on health by regulating outcomes, law—understood as encompassing issues of governance—plays a role in regulating the behaviour of powerful actors.^45^ It regulates government and non-government institutions; that is Local Planning Authorities, the Planning Inspectorate, and branches of government responsible for, or having an impact on, urban development, such as the Department for Levelling Up, Housing and Communities, the Department for Transport, or the Department for Environment, Food and Rural Affairs. Different types of specific laws govern the good functioning of powerful institutions and industries. In England, private actors such as developers, real estate investors, and development corporations, as well as non-governmental bodies such as the Environment Agency and Natural England, exercise power over what gets built and how.^46^ As a result, any law governing these entities has the potential of limiting, or at least controlling, their power to protect the social determinants of health. This brings us back to the idea that the law regulates, in part, the distribution of power, money, and resources in our society, including powerful private and public actors in urban development.

The direct and indirect effect of laws on health in urban development, together with the multiplicity of government and non-government actors, as well as law taken as structural rules organising our society in a capitalist market economy, are all different faces of the law acting as a key determinant of health. In this section, I have demonstrated how law, both as a concept of structural organisation, daily rules, and as a concept of governance of actors, shapes the daily conditions in which we are born, grow, work, live, and age; that is, the social determinants of health. I will now turn to human rights theory and discuss why human rights are relevant to researching the causes of ill health in urban development.

### C. Why human rights are relevant

I argue in this section that fundamental human rights principles of universalism, interrelatedness, and indivisibility mean that when we talk about the social determinants of health, we are talking about human rights. *A contrario*, human rights and health cannot be researched without consideration of the social determinants of health because public health and human rights share a common goal of promoting human well-being. I turn to these two points below, before discussing how the right to health helps understand the links between public health and human rights principles in urban development.

#### 1. Universalism, interrelatedness, and indivisibility of human rights

Universalism may be understood at various levels. At the fundamental level, universalism stems from the inalienability of human rights; that is, that everyone is entitled to human rights by virtue of being human.^47^ However, as Jack Donnelly has argued, the fact that human rights are universally *held* does not show that such rights *exist* universally. Donnelly shows that human rights, although conceptually universal, are not enforced universally, nor are they understood universally at historical and anthropological levels. However, even for those who do not accept that human rights are fundamentally universal, Donnelly provides an account of what he calls ‘relative universalism’. He grounds human rights into a functional universality, to provide a practical answer to modern threats to human dignity:

Human rights today remain the only proven effective means to assure human dignity in societies dominated by markets and states. Although historically contingent and relative, this functional universality fully merits the label universal – for us, today.^48^

Universalism of human rights, whether understood conceptually as ‘inalienability’ or functionally to protect human dignity in our globalised world, suggests that human rights are interrelated and so taking one single human right in isolation makes little sense. To extend Donnelly’s reasoning, conceptually, dignity stemming from ‘being human’ cannot mean that only a few human rights are relevant to being human. All human rights apply to us by virtue of being human. In a similar fashion, functionally, human rights would lose a great deal of their power if they were conceived independent from one another. Universalism, or even ‘relative universalism’ as argued by Donnelly, requires us to think of human rights as interrelated.

The so-called ‘interrelatedness’ of human rights is a fundamental principle reaffirmed in paragraph I.5 of the Vienna Declaration and Programme of Action.^49^ From a practical perspective, as posited by the United Nations, this means that violation of one right results in conditions that lead to the violation of other rights.^50^ For instance, violation of the right to housing, such as living in a poor-quality home with dampness, creates conditions in which the right to health cannot be fulfilled. Similarly, violation of the right to health, because of unaffordable health care or unreasonably long distances of travel to access care, creates conditions where a person’s employment is put at risk, thereby potentially leading to violation of the right to work. On a more general level, violation of economic and social rights, such as housing, work, health, and education, creates conditions in which civil and political rights, including freedom of assembly, freedom of speech and thought, and right to participate in political life, cannot be realised fully, and vice versa.^51^

The interrelatedness of human rights echoes research on the social determinants of health which shows that the daily conditions in which we live have an effect on the distribution of health in the population. These daily conditions are, in fact, our economic and social rights to housing, education, work, water, and health, as well as our civil and political right to freedom of thought and to participate in political life.^52^ The WHO Commission’s report discusses 12 areas as key drivers of the unequal distribution of health, of which seven at least can be linked to human rights (Table 1).

**Table 1.** Synergies between the social determinants of health and human rights

Social determinant of health^a^	Corresponding human right(s)	Corresponding provision(s) in international human rights law
Early child development and education	Right of the child to the enjoyment of the highest attainable standard of health and to facilities for the treatment of illness and rehabilitation of health Right of the child to education	Article 24, Convention on the Rights of the Child (CRC)^b^ Articles 28 and 29, CRC
Healthy places and the living environment	Right of everyone to the enjoyment of the highest attainable standard of physical and mental health (right to health) Right to protection of health Right to a healthy environment	Article 12, International Covenant on Economic, Social and Cultural Rights (ICESCR)^c^ Article 11, European Social Charter^d^ UN Resolution recognising a Human Right to a Healthy Environment^e^
Fair employment and decent work	Right to work Right to just and favourable conditions of work	Article 6, ICESCR Article 7, ICESCR Articles 1–10, European Social Charter
Social protection across the life course	Right to health Right to protection of health Right to social security Right to work	Article 12, ICESCR Article 11, European Social Charter Article 12, European Social Charter Article 6, ICESCR
Universal health care	Right to health Right to protection of health	Article 12, ICESCR Article 11, European Social Charter
Gender equity	Women’s rights States’ duty of non-discrimination when realising economic, social, and cultural rights Equal rights of men and women to the enjoyment of all economic, social, and cultural rights	Convention on the Elimination of All Forms of Discrimination Against Women^f^ Article 2.2, ICESCR Article 3, ICESCR
Political empowerment	Civil and political rights	International Covenant on Civil and Political Rights^g^

a Michael Marmot and others, ‘Closing the Gap in a Generation: Health Equity Through Action on the Social Determinants of Health’ (2008) 372 The Lancet 1661.

b UN General Assembly, Convention on the Rights of the Child, 20 November 1989, United Nations, Treaty Series, vol 1577, p 3.

c UN General Assembly, International Covenant on Economic, Social and Cultural Rights (ICESCR), 16 December 1966, United Nations, Treaty Series, vol 993, p 3.

d Council of Europe, European Social Charter (Revised), 3 May 1996, ETS 163.

e UN Resolution A/HRC/48/L.23/Rev.1.

f UN General Assembly, Convention on the Elimination of All Forms of Discrimination Against Women, 18 December 1979, United Nations, Treaty Series, vol 1249, p 13.

g UN General Assembly, International Covenant on Civil and Political Rights, 16 December 1966, United Nations, Treaty Series, vol 999, p 171.

The principle of interrelatedness of all human rights means that every human right is equally important. One right cannot and should not be more valued than another, and it would make little sense, conceptually and practically, to compare one right to another. This is what is referred to as the principle of ‘indivisibility’ of human rights.^53^

In summary, when the social determinants of health are examined, human rights cannot be ignored because, in fact as well as in principle, the social determinants of health are *about* human rights.^54^ Equally, it is not possible to research the right to health whilst ignoring social epidemiological evidence on the social determinants of health.^55^

#### 2. The common goal of public health and human rights

In addition to being intricately linked to the social determinants of health, human rights share a common goal with public health. In 1994, Jonathan Mann and colleagues identified three conceptual links between health and human rights, which remain valid and relevant today: the impact of health policies on human rights; the health impacts resulting from human rights violations; and an inextricable linkage between health and human rights due to, *inter alia*, the ‘central problem of defining and advancing human well-being’.^56^

The common goal shared by public health and human rights, as well as the complementarity of approaches that they offer to protecting and promoting well-being, are a solid basis for including human rights in research on urban development and health. By way of illustrating how human rights are relevant to this issue, I will now consider the right to health itself.

#### 3. The right to health

Health is a human right enshrined in Article 25 of the Universal Declaration of Human Rights, and in binding international human rights treaties, principally Article 12 of the International Covenant on Economic, Social, and Cultural Rights (ICESCR).^57^ The right to health is also integrated into regional human rights instruments, such as Article 11 of the European Social Charter, to which the UK is a signatory.^58^ In 2000, the content of the right to health was clarified with the publication of General Comment 14 by the Committee on Economic, Social and Cultural Rights (CESCR), the body responsible for overseeing the implementation of the ICESCR.^59^ General Comment 14 interprets the right to health as extending to ‘the underlying determinants of health, such as food and nutrition, housing, access to safe and potable water and adequate sanitation, safe and healthy working conditions, and a healthy environment’.^60^

The underlying determinants of health share considerable resemblance with the social determinants of health. The First Special Rapporteur on the right to health, Paul Hunt, recommended that the right to health should be understood as part of a national health system, which itself is part of a broader social structure influenced by political and economic considerations.^61^ This conceptualisation of the right to health as being influenced by, or even the result of, our economic, social, and political system, shows similarities with the ‘causes of causes’; that is, issues of power, money, and resources as formulated in social epidemiology.

With this in mind, tackling the causes of unhealthy urban development necessitates not only consideration of the social determinants of health and the role of the law in shaping them, but should also draw from human rights to inform both health research itself and practical solutions to preventing NCDs and reducing health inequalities. Having defined the scope of the research, in the second part of this article I will now apply the tools that the law, including international human rights law, offers, as well as discussing the value and limits of using a human rights framework in urban development and health.

## III. APPLICATION OF THE LAW AND HUMAN RIGHTS TO URBAN DEVELOPMENT AND HEALTH

In Section II, I demonstrated how the social determinants of health provide a solid basis to the study of health in the context of urban development. The rich and longstanding research on the social determinants of health shows that the daily conditions in which we live are a determining factor of the unequal distribution of health in society. In turn, these conditions are shaped by the unequal distribution of power, money, and resources, factors for which the law is a key determinant. By conceptualising the law in a broad sense, encompassing the governance of powerful institutions and by considering it as part of wider socio-political phenomena, the study of law may shed light on the root causes of social inequalities and so on the causes of unequal distribution of health. Furthermore, the laws shaping urban planning belong to a multiplicity of areas within the law itself, including administrative law, procurement, environmental law, planning law, and building regulations. Given this, I now turn to the question of what mechanisms of law ought to be studied in this context.

The Lancet-O’Neill Commission on Global Health and the Law reported on the impact of the law on health outcomes, looking at subnational, national, and inter- and supranational levels in four areas: (i) translating vision into action; (ii) strengthening the governance of national and global health institutions; (iii) implementing fair, evidence-based health interventions; and (iv) building legal capacities for health.^62^ I rely on these four legal determinants to illustrate what mechanisms of the law ought to be deployed in urban development to protect and promote health. I then apply a human rights framework to these mechanisms, before discussing the value and limits of this approach.

### A. Mechanisms of the law to promote health in urban development

#### 1. Translating vision into action

Setting clear objectives and corresponding principles is needed to guide decisions within a sector—such as urban development—but also across sectors; for example, planning, public health, and the economy. Although the NPPF mentions health as a component of its core objective to achieving sustainable development, key stakeholders, such as planning officers and developers, lack guidance on how to implement this vision.^63^ For instance, does promoting health provide grounds to refuse a planning application of a new housing development without any community garden, or with small spaces, or with poor quality housing? What can be considered, legally, poor quality housing? And how does it compare with the need to provide large numbers of accommodations? Does promoting health provide enough grounds to refuse a planning application for a car park, and in what circumstances? How does health weigh against material considerations that, legally, must inform decisions?^64^

The NPPF specifies that its objective of achieving sustainable development and, within that, to ‘support communities’ health, social and cultural well-being’, does not constitute a criterion to base decisions on.^65^ Rather, this objective should only inform the design of local development plans, their implementation, and planning authorisations, whilst taking into account other factors such as local circumstances.^66^

As the Lancet-O’Neill Commission indicates, ‘law provides both the mandate for [a goal] and the tools to achieve it’.^67^ Providing the mandate for promoting health and reducing health inequalities could become a clear goal of the NPPF and of local development plans informing planning decisions in England. At first, explicitly stating this goal may appear at odds with the complex decision-making environment that defines urban planning in England. Health may be seen as one of many goals of urban planning, such as promoting a strong economy and delivering a targeted number of houses each year.^68^ Nevertheless, health is a central value to society, and given the critical determining action of urban development on health, it appears as a sufficient reason to uphold it as (one of) the key goal(s) of urban development. This vision conceptualises health as the core value for our society.^69^

Stating health as a main and explicit goal of urban development would be in line with the ‘health-in-all-policies’ agenda of the WHO.^70^ Including health in all policies and beyond strictly the health sector is necessary to ensure cohesion in all areas to protect and promote health. Venkatapuram argues that this concern for health in all sectors is imperative to tackling the unequal distribution of economic and social conditions.^71^ To some extent, the proposed Healthy Homes Bill—being read in the House of Lords at the time of writing—would implement a vision promoting health in new homes in the UK through the implementation of the ‘healthy homes principles’.^72^

Nevertheless, making health promotion, or even the reduction of health inequalities, a clear goal of urban development is not sufficient. In addition to providing a mandate, the law has the capacity to provide the tools to achieve a goal.^73^ Taking the case study of universal health coverage, the Lancet-O’Neill Commission shows how the law may be of value in assisting governments making tough policy choices:

Law and regulation are integral to making difficult choices because they establish processes and institutions to guide transparent decision-making. States use legal processes to express national health priorities, and legal frameworks define and delineate that which is possible for a state to achieve.^74^

The use of the law as a guide to making policy choices is directly applicable to the context of urban development. In fact, once health priorities have been set out clearly at the national level, including in the NPPF, local development plans constitute the legal framework that define and delineate what is possible to achieve in any given urban context. By establishing clear standards that inform new local development plans, neighbourhood plans, and planning authorisations, Local Planning Authorities will be equipped to make decisions that are in line with the goal of promoting health and reducing health inequalities in urban contexts, and that are consistent between regions and over time. These standards must be discussed by experts in several disciplines, including urban development, health, and economics, in a transparent and participatory fashion.

Welsh legislation is one example of how vision can be implemented into action and provide guidance on public policy. The Well-being of Future Generations (Wales) Act 2015 implements core principles of the United Nations Sustainable Development Goals, including promoting physical and mental health, building places resilient to climate change, and improving equality in society.^75^ Under this law, public bodies in Wales are subject to a well-being duty, meaning that they must set clear objectives to implement the seven principles of sustainable development and take action to meet these objectives. The Act set five core ways of working, including collaboration, involvement of communities, and long-term impact. It also created implementation mechanisms. For instance, Public Services Boards foster collaboration between local authorities and other public bodies in a given area. A Future Generations Commissioner conducts research, assesses, and advises public bodies on their well-being objectives and progress towards them. The Auditor General evaluates public bodies’ actions against the objectives set. The effectiveness of these compliance mechanisms has been questioned, notably due to the use of non-mandatory terms in the Act, as well as dependency on how the powers of the Future Generations Commissioner are exercised.^76^ Nevertheless, the Act remains the first legislation in the world to impose a legal duty on public bodies to respect the well-being of future generations. By enunciating clear sustainable goals, creating implementation and monitoring mechanisms, and setting ways of working, the Act contributes to implementing the core vision of sustainable development in practice.

#### 2. Strengthening governance of national institutions, local planning authorities, and powerful private organisations

The Lancet-Oslo Commission on Global Governance for Health, which preceded and informed the Lancet-O’Neill Commission, defined global governance as:

The complex of formal and informal institutions, mechanisms, relationships, and processes between and among states, markets, citizens, and organisations, both intergovernmental and non-governmental, through which collective interests on the global plane are articulated, rights and obligations are established, and differences are mediated.^77^

Although this definition was written in the context of *global* governance, its characteristics apply to national governance of urban development and health; that is, as a diffuse concept encompassing a broad range of actors, processes, and forces.

Good governance is critical to a well-functioning State and the implementation of the law.^78^ In contrast, issues of corruption, whether at national or local levels, undermine transparent decision-making and divert taxpayers’ money away from social functions.^79^ The Lancet-O’Neill Commission identified three governance challenges in global health, of which two are applicable in the context of this research.^80^ First, health in urban development in England, similar to global health, is characterised by ‘fragmented and overlapping mandates of actors and institutions’.^81^ Indeed, several governmental departments may enact policies or programmes with a direct impact on urban development; for example, the Department for Levelling Up, Housing and Communities, the Department for Transport, or the Department for Environment, Food and Rural Affairs. At the local level, Local Planning Authorities design new local development plans and decide to grant or refuse planning applications. Their mandates sometimes overlap with a regional authority. For example, some questions of transport, land development, and housing at Bristol City Council must be discussed with the West of England Combined Authority.^82^ In addition, private actors, such as developers and real estate investors, have an impact on what gets built and how, because they design new projects and apply for permission to build them. At the national level, the Royal Town Planning Institute (RTPI)—the Chartered Institute and membership organisation in urban planning—has considerable influence on setting and maintaining professional standards. The RTPI website indicates that its areas of influence span from housing, transport and infrastructure, climate change in urban planning, to the economy, and health and inclusive planning. The scattered and multilevel governance of these public and private actors creates unfavourable conditions to implement a shared vision of health, let alone the right to health. Law can be used as a tool to clarify functions in relation to population health and encourage cooperation to enable coherent practices that, ultimately, promote health and reduce health inequalities.^83^

The second challenge identified by the Lancet-O’Neill Commission relates to ‘weak monitoring, compliance, and enforcement’.^84^ To take one example, weak enforcement in building safety affects health and may lead to potentially disastrous consequences in urban development. The Grenfell Tower disaster is one extreme example of this, but, more generally, health consequences of poor building conditions can be observed,^85^ such as the association between dampness or mould and increased risks of asthma. Law has the capacity to establish strong enforcement mechanisms, including by creating and governing dispute resolution bodies such as the New Homes Ombudsman, as well as mandating independent agents to check compliance during and post-construction.

#### 3. Implementation of evidence-based interventions

Law is a means to implement interventions that we know scientifically work in any given context. In England, urban planners and developers may not be fully aware of the harmful impacts of planning measures on population health,^86^ but it is important to first assess whether evidence of these impacts exists, or whether, whilst published in scientific journals, it is not known of by practitioners. If that is the case, efforts should be directed at bridging the gap between science and practice.

Law may be used as a powerful means by policy-makers at national and local levels to implement ‘what works’ and guard against measures that are harmful to population health and deepen health inequalities.^87^ For example, if sound scientific studies show that car parks located near schools worsen asthma in children, or that ‘no traffic zones’ increase healthy and green modes of transport such as walking and cycling, then such measures could be implemented by law. Evidence may show ‘what works’ in a specific urban context; for example, cycle lanes may not be appropriate in neighbourhood with high levels of street crime. In this case, it is important that local policy-makers look at what evidence is available in the context of their own city/region, or conduct impact assessments of measures that are suggested in another context to assess whether they would have the desired impact in their own local area.

Lack of evidence of positive impact on health should not be an excuse to delay or deny the implementation of measures that may be beneficial to health.^88^ This is especially true in the context of urban planning and health research, where causation at the population level is quasi-impossible to establish due to the number of variables confounding the outcome, the time between implementation of any given policy or programme and its impact, and the long latent period in NCDs between exposure to a harmful agent and observation of the first symptoms. The Lancet-O’Neill Commission pre-empted this potential policy inertia:

While using the best available (albeit incomplete) evidence at the time, evaluating the law’s effect to ensure continuous quality improvement in health legislation is imperative. Even then, though, law makers might need to accept some uncertainty, such as where there is clear evidence that a suite of legal measures is effective at addressing a complex health threat (e.g., obesity, or gun violence), but it is difficult to determine the specific contribution of any single intervention.^89^

With regard to those health policies that we know work, the WHO has published a list of ‘best-buys’ for public health; that is, cost-effective and evidence-based.^90^ Such laws and regulations are found in the WHO’s Global Action Plan for the prevention and control of NCDs 2013–2020.^91^ Some of the measures mentioned can be implemented using urban planning tools. For instance, ‘eliminating exposure to second-hand tobacco smoke in all indoor workplaces, public places, public transport, and in all outdoor mass-gathering areas’ may be implemented by Local Planning Authorities via the designation of non-smoking zones in the city, such as near schools and shopping centres.^92^ Furthermore, zoning and the designation of building uses may contribute at the local level to ‘restrictions on physical availability of retailed alcohol’.^93^ Two measures are directly implementable via Local Planning Authorities: ‘Ensuring that macro-level urban design incorporates the core elements of residential density, such as connected street networks that include sidewalks, easy access to a diversity of destinations, and access to public transport’, and ‘Providing convenient and safe access to quality public open space and adequate infrastructure to support walking and cycling’.^94^ Such measures would promote cities that are ‘socially cohesive’ and ‘ensure access to basic goods’ in England and elsewhere, and therefore ultimately promote health equity.^95^

#### 4. Building and strengthening legal capacities for health

The final legal determinant discussed in the Lancet-O’Neill Commission report is legal capacity building, with action in three areas:

Legal capacity for health refers to three interlinked dimensions: effective legal environments (which include the infrastructure for drafting, implementing, and enforcing laws that promote health with justice, as well as fairly resolving grievances that arise); a strong and growing evidence base, built on the rigorous monitoring and evaluation of existing laws; and an empowered transdisciplinary health law workforce. The latter includes connected networks of well-trained professionals – legal and non-legal – who share information and strategies, and who provide technical legal assistance.^96^

The first two areas are directly relevant to urban planning. As for the third, the argument may be extended to legal capacity building and awareness of *health and the law* in *urban planning*. Legal capacity could be strengthened to empower decision makers in Local Planning Authorities with sound legal knowledge so that they have the confidence to make decisions in favour of population health. Some institutions are already in place to deliver courses and training to practitioners, such as the RTPI. This function may be used to train Local Planning Authorities, developers, real estate investors, and the Planning Inspectorate to consistently promote health in planning decisions, and to recognise the circumstances under which a new development project would harm health and/or deepen inequalities.

In summary, the law may be deployed in (at least) four ways to promote health and reduce health inequalities in urban development in England (and elsewhere). I have focused on the legal determinants discussed in the Lancet-O’Neill report as a basis for my reflection, and I will now extend this to human rights.

### B. Application of a human rights framework to urban development and health

As mentioned in Section II above, human rights theory and practice complement public health approaches. Here, I explore how a human rights framework may add analytical power to the legal determinants of health and fill some gaps. In particular, I discuss the first two legal determinants mentioned above and argue that human rights principles provide guidance in the implementation of policy coherence and good governance in urban planning. I will then show how a human rights framework might promote a change in the way health is conceived and interpreted by powerful actors in urban planning in England.

#### 1. Legal determinant 1 and the human rights analytical framework

Human rights may complement the role of the law in making policy choices. Whilst a human rights framework is certainly not the only tool to facilitate choices in public policy, it may contribute to making such choices. A human rights framework, or rights-based approach, sets out clear principles that must guide policy objectives and decision-making at all levels. This analytical framework originated with General Comment 4 on the right to housing and has been applied to other human rights with slight variations, for instance, the right to food, the right to education, and the right to health.^97^

In the context of urban planning, the framework as applied to the right to housing could constitute a solid basis to ground policy objectives into human rights principles, and to assist officers in Local Planning Authorities to grant or refuse planning permissions. In General Comment 4, the CESCR recognises the principles of legal security and tenure; availability of services, materials, facilities, and infrastructure; affordability; habitability; accessibility of housing to disadvantaged groups; location; and cultural adequacy of housing.^98^ Whilst it is not possible to discuss all these principles in detail, I consider three of them below and explain how they could inform decisions in urban development in England: affordability, habitability, and locaton.

First, the principle of ‘affordability’ of housing is defined by the CESCR as:

Personal or household financial costs associated with housing should be at such a level that the attainment and satisfaction of other basic needs are not threatened or compromised. States parties should take steps to ensure that the percentage of housing-related costs is, in general, commensurate with income levels. States parties should establish housing subsidies for those unable to obtain affordable housing, as well as forms and levels of housing finance which adequately reflect housing needs. In accordance with the principle of affordability, tenants should be protected by appropriate means against unreasonable rent levels or rent increases.^99^

This interpretation of ‘affordability’ might guide policy in what constitutes ‘affordable housing’ in England. For instance, under such a principle it would not be tolerable for developers to fulfil the condition of affordability by offering shared ownership of above market-priced new-built accommodations.

In addition, General Comment 4 explains the principle of ‘habitability’ as:

Adequate housing must be habitable, in terms of providing the inhabitants with adequate space and protecting them from cold, damp, heat, rain, wind or other threats to health, structural hazards, and disease vectors. The physical safety of occupants must be guaranteed as well.^100^

The CESCR refers to the WHO Health Principles of Housing as a guide to promote adequate conditions of habitability.^101^ Since General Comment 4 was written, other international guidance may have been published on what constitutes adequate standards of habitability, especially under the mandate of UN Habitat, which could inform decisions in urban planning. For instance, Local Planning Authorities may need to refurbish, or in a last resort, demolish and replace, current social housing in poor condition before accepting planning applications for a new luxury housing project.

Interestingly, General Comment 4 adds to the framework the principle of ‘location’:

Adequate housing must be in a location which allows access to employment options, health-care services, schools, childcare centres and other social facilities. This is true both in large cities and in rural areas where the temporal and financial costs of getting to and from the place of work can place excessive demands upon the budgets of poor households. Similarly, housing should not be built on polluted sites nor in immediate proximity to pollution sources that threaten the right to health of the inhabitants.^102^

Whilst the connectivity between communities and between new projects and town centres is already informing decision-making in urban planning, the level of pollution may not be taken into account systematically.^103^ The principle of location would mean that a newly built scheme in a busy area of a city with high levels of traffic should not be accepted until pollution levels are lowered. It would also mean that Local Planning Authorities have a duty to reduce traffic levels near residential areas. In this regard, the 2020 decision issued by the Coroner for London Inner South linking an individual death to air pollution is fully in line with a human rights-based approach to housing and urban planning, and may constitute a first step towards a new legal conceptualisation of accountability of the State with regard to air pollution and health.^104^ Some principles of a human rights-based approach to housing are incorporated in the new Healthy Homes Bill for England and Wales, such as a minimum liveable space (principle b), access to natural light (principle c), inclusive, accessible and adaptable places for all, taking into account protected characteristics under the Equality Act (principle d), access to sustainable transport and walkable service (principle e), or reduction in carbon emissions and resilience to climate change (principles f and g). At the time of writing though, these principles would apply to new homes only.

Taken together with the view that law provides the tools to translate vision into action, these principles may constitute a solid basis to guide the NPPF, local development plans, and to inform decisions to grant or refuse planning permissions by Local Planning Authorities.

#### 2. Legal determinant 2 and the right to a process

Human rights can contribute to using the law as a tool of good governance to regulate behaviours and relationships of powerful governmental and non-governmental institutions having an impact on health in urban development. The principles of accountability, transparency of decision-making, participation of affected communities, and non-discrimination are well defined in human rights, with guidance on how to implement them. These principles form a right to a process applicable to all economic and social rights, including the right to health.^105^ Non-discrimination (Article 2.2 ICESCR) and equality between men and women (Article 3 ICESCR) are overlapping principles that apply to all the rights protected by the ICESCR, including the right to housing and the right to health.^106^ The application of human rights on a non-discriminatory manner is reiterated in many General Comments, including General Comment 4 on the right to housing and General Comment 14 on the right to health.

In practice, a human rights framework would include non-discrimination as a core principle enshrined in the NPPF to guide decisions of Local Planning Authorities. The principle may even extend to private actors such as developers and real estate investors when it is recognised that such actors perform a public function; for example, building housing necessary to reach the housing target of a local area, or building affordable housing.

In England, Scotland, and Wales, the Equality Act 2010 implements non-discrimination in national law.^107^ The Act prohibits discrimination on ‘protected characteristics’ which are similar in scope to grounds of discrimination in international human rights law.^108^ The equality duty applies to public bodies as well as private organisations carrying out public functions, which provides a solid basis, in national law, to implement equality and non-discrimination as principles to guide decisions in urban development. Likewise, Article 14 of the European Convention on Human Rights, enforceable in the UK under the Human Rights Act 1998, protects against discrimination in the enjoyment of Convention rights.^109^ Of particular interest to planning decisions, potential indirect discriminatory effects of a policy or decision by a public authority are also protected under section 19 of the Equality Act.^110^ Section 6(1) of the Human Rights Act adds that a public authority cannot act in a way that is incompatible with a right protected by the Act, including non-discrimination.^111^ Despite these domestic protections, it is not clear how much the equality duty and the provision against discrimination weigh in decisions taken by Local Planning Authorities to grant or refuse planning permission, compared to other priorities such as the financial viability of a project. Integrating the equality duty and provisions against direct and indirect discrimination in decision frameworks of Local Planning Authorities would offer additional guidance, in particular with regard to the actual effects of new development projects on increasing or decreasing social inequalities and their related impacts on health.

In parallel to non-discrimination, accountability of institutional actors in urban development may equip such institutions with processes to scrutinise decisions and means to redress inequalities. Accountability means that States, including their organisations and agents, are primary duty-bearers, and citizens are rights-holders.^112^ Meaningful accountability is not about shaming;it is about the ability of citizens to participate in, and scrutinise, decisions that affect them, as well as to obtain redress when such decisions result in violations of their rights. As such, real accountability means implementing the principles of transparency, participation, and access to remedies. The Human Rights Act 1998 offers judicial remedies when an act of a public authority breaches the rights protected by the Act, including both monetary and non-monetary compensation.^113^ This reflects international human rights law where remedies do not necessarily equate to financial compensation, but also to satisfaction, restitution, and guarantee of non-repetition to alleviate a situation where rights are violated.^114^ However, judicial remedies—although necessary—are insufficient alone because they require the affected person(s) to bring legal action, something that people who lack time, knowledge and money may not be able to do. With regard to the right to health, Alicia Ely Yamin has argued that accountability of duty-bearers is about what the State is doing; how much effort it makes towards the realisation of the right to health (progress); and how it implements the right ( process).^115^ I posit that this conceptualisation of accountability may be extended to the context of preventing NCDs and reducing health inequalities in urban development. Accountability would mean scrutinising planning decisions on the basis of whether they advance health and reduce social inequalities, for example, as is now the case in Wales with the Future Generations Commissioner.^116^ Such concerns would be weighed against other priorities, in particular the financial viability of a project. In order to assess meaningfully health (dis)benefits against, say, financial viability, data would need to inform the projected impacts on health and social inequalities, in the same way that financial data is included to determine the viability of a project.

Similarly, a human rights process would require active and meaningful participation of affected communities. Public consultation already forms an integral part of urban planning projects; for example, with nationally significant infrastructure projects.^117^ However, it may be reduced to a simple box-ticking exercise when not conducted meaningfully because affected communities may not have the time to participate in such consultations, or they may not even realise that a public consultation is underway for a specific area in their town. In addition, a disproportionate number of people coming from disadvantaged communities may not be equipped to understand the legal jargon used in planning applications. Sometimes, the consultation may be conducted by a consultant whose priority is to foster good business relations with the developer. It may be conducted too late, after the project is already decided, which effectively strips communities from their voice. A meaningful consultation should be conducted by an independent third party, well ahead of a project, with information communicated in a clear and concise format, with data showing potential impacts of the project, including on health and inequalities, and shared openly. If a consultation requires affected communities to attend discussions such as workshops, offering financial compensation when people are obliged to take time off work would encourage, and may increase, participation of populations who would not usually be involved in such consultations.

These examples illustrate how human rights principles of non-discrimination, accountability, transparency, participation, and redress can be implemented to ensure good governance of public and private actors and guide their actions and decisions in urban development. This right to a process would ensure better health promotion and reduction of health inequalities. It may also contribute to debates on making choices in public policy by stating which values ought to be prioritised and through which principles they should be implemented, something that the Well-being of Future Generations Act aims to assure in Wales. Beyond the application of human rights principles to the legal determinants identified in the Lancet-O’Neill report, using human rights may empower communities and generate systemic change.

#### 3. Social mobilisation

Yamin and Andrés Constantin have noted the power of human rights as both legal claims and discourses for social mobilisation.^118^ In the context of urban development and health, empowered citizens would be more likely to act upon harmful decisions, using either their voice to show discontent through the courts challenging a planning permission of a new project, or claiming protection of their human right to health, right to housing, right to life, or right to a healthy environment. The State may contest these types of action. For example, the reduction in England of the time limit from six to three months to file a judicial review claim substantially reduced the financial and material means of civil society organisations to contest the planning of large new infrastructure projects.^119^

Nevertheless, applying human rights has multiple advantages to furthering health in urban development, in particular by analysing laws and policies, implementing good governance, as well as empowering communities and mobilising social movement. Yet, human rights theory and practice face their own limits, and will not solve all issues in urban development and health.

### C. The added value and limits of a human rights approach

Before discussing the limits of a human rights framework, it is worth stating the benefits that such an approach conveys. Using human rights language turns moral values (for example, health) into legal entitlements (the right to health as protected by, *inter alia*, Article 12 ICESCR). Philip Alston expressed this idea in a compelling manner:

Anyone who has spoken with development economists, government officials or experts from international agencies about economic and social rights will almost certainly have been told: ‘What does it matter if we call something a right, a need, a goal, an objective, or a target? It makes no difference, since we all want the same result!’ The reality, of course, is that it makes a world of difference. Needs can be deferred until those in power think it might be timely to address them. Needs can be defined and formulated by experts; they are usually seen to be eminently flexible and relative …. Rights, on the other hand, belong to individuals, who can and will assert them and strive to give them meaning and substance. They can be neither expropriated, nor defined, nor arbitrarily put on the back burner, by officials.^120^

Referring to claims as human rights gives them legal and moral legitimacy, enhances coherence in policy-making and practice across different sectors, and, therefore, improves effectiveness of the policy-making and implementation.^121^ This is particularly relevant in the context of urban development, where competing objectives, short political cycles, and limited resources convolute decisions in Local Planning Authorities.

However, using human rights may face important conceptual—and arguably practical-limits. Audrey Chapman discussed the application of human rights theory to the social determinants of health in an article published in 2010 that is still relevant today.^122^ First, the conceptualisation of equality differs between human rights and social epidemiology. Chapman argued that whilst human rights are concerned with equality of everyone before the law (formal equality), the social determinants of health are concerned with substantive equality in society. The principle of non-discrimination in human rights could require both formal and substantive non-discrimination, in the letter of the law as well as in its effect - intended and unintended,^123^ and this broad understanding of discrimination is embedded into UK law in section 19 of the Equality Act 2010.^124^ However, this is quite different from substantive equality at all levels of society as a foundational principle of research on the social determinants of health.^125^ In other words, the social determinants of health aim to achieve equal societies, whereas human rights are targeted to prevent and remedy discriminatory use of the law and State power.

The reach of measures or social action recommended by both approaches also differs. In human rights law, action should focus on the most vulnerable groups of society as a matter of priority, whilst the social determinants of health recommend action to remedy unequal societies in order to reduce social inequalities at a general level and in all health gradients for everyone.^126^ In British urban development, using the concept of equality as defined in social epidemiology might help to better target health inequalities at all levels of society and prevent risks of NCDs in the future, and have an impact on the population as a whole. However, resource constraints in Local Planning Authorities mean that difficult choices must be made, and that vulnerable communities should be prioritised. In this case, a human rights approach to non-discrimination, although not perfect, may be better suited to guide public policy decisions.

In addition, Chapman found that the scope of action in human rights is narrower than the social determinants of health. She referred to the ‘underlying determinants of health’ as enunciated in General Comment 14 and reports of the Special Rapporteur on the right to health, and showed that this term refers principally to the conditions necessary to be in good health, such as clean water and sanitation, adequate housing, adequate supply of nutritious food, or health-related education and information.^127^ In contrast, the social determinants of health reach to daily conditions extending to the availability and quality of education, fair employment conditions, political empowerment, urban planning, as well as issues of distribution of power, money, and resources.^128^ In that regard, the underlying determinants of health, as defined in international human rights law documents, would have a narrower impact than the social determinants of health in urban planning. To some extent, the underlying determinants already inform urban planning decisions; that is, provision of clean water and sanitation, or adequate supply of food in a local area. Public bodies may, though, benefit from implementing a vision of the social determinants of health to guide their objectives and decisions. For example, such a vision may encourage a fairer distribution of power and resources between central government and local authorities.

This last remark reveals the limits of a human rights framework to address the prevention of ill health and amelioration of health inequalities in urban development. As demonstrated above, and with rich and compelling research on the social determinants of health, the unequal distribution of power, money, and resources lies at the heart of the interrelated problems of poor health outcomes and health inequalities. However, it is unclear whether human rights aim to, and indeed do, address systemic issues of power in market-driven societies. Whilst I admit that human rights cannot be apolitical, the process of their implementation and monitoring seems to avoid, as much as possible, political disputes.^129^ This may be due, in part, to the fact that the implementation and monitoring of human rights are largely constrained by State sovereignty. Some may even argue that the implementation of human rights is dependent on States’ goodwill.^130^

Despite these limits, there are important benefits of a human rights framework, as I have sought to demonstrate here, and may contribute to debates on integrating values, such as health equity, in public policy and decision-making processes. For this reason, I concur with the thinking of human rights scholars who argue that a human rights approach, or, more specifically, the right to health, should be more concerned with addressing structural issues of power distribution in market capitalist economies.^131^ As Yamin and Constantin said in their review of the health rights from the 1990s to 2017:

[I]n order to fulfil their promise to change the systems that perpetuate inequality, human rights-based approaches to health ‘must adopt a holistic and integrated approach to health systems’ and examine ‘the dominant assumptions underlying the structural determinants of … health … [including] strategies to address those factors, to reshape the possibility frontier for advancing … health.’ In other words, they challenge power structures and the status quo. A human rights-based approach therefore changes decision-making processes and the issues and actors included in those processes, as well as outcomes.^132^

## IV. CONCLUSION

In this article, I have explored the conceptual and practical links between urban development, health, law, and human rights. I grounded the analysis in literatures across the disciplines of global health, law, and human rights. Using research on the social determinants of health as a basis, I have shown how law, conceptualised in a broad sense, is a key determinant of health. I argued that human rights—as enacted in international human rights law and partially implemented in UK domestic legislation—are relevant to research in health and urban environments. Principles of universalism, interrelatedness, and indivisibility essentially mean that the social determinants of health are about human rights. Furthermore, human rights share a common goal with public health, ie promoting human well-being.

I relied on the Lancet-O’Neill report to show how the power of the law may be deployed to promote health and reduce health inequalities in urban development in England. I supplemented this analysis by explaining how a human rights framework may provide additional guidance, in particular by implementing non-discrimination, equality, accountability of the State and its agents, participation of affected communities, transparency of decisions, and redress. Although such principles are found in international human rights law, the principles of non-discrimination and equality have the added value of being justiciable in England through the Equality Act 2010 (in England, Scotland, and Wales) and the Human Rights Act 1998. Finally, I discussed the limits of a human rights framework, especially in relation to the consideration that they have for structural inequalities of power, and I argued that more attention should be paid to the ‘causes of causes’.

This article adds to the literature on two levels. First, conceptually, it explores the links between interrelated questions in public health, law, human rights, and urban planning to achieve the common goal of human well-being. In doing so, I have applied the global health concept of the legal determinants of health to the domestic context of English urban development. Secondly, this research offers practical suggestions on the use of a human rights framework to determine health as a core value of urban development to achieve public policy goals. Whilst contributing to debates on competing priorities in public policy, it highlights the need for further research in the problem of empowering regulators and decision makers effectively to balance competing public policy goals in urban development. This type of research should be informed by several disciplines, including law, urban planning, public policy, public health, and human rights, and be complemented, as far as possible, by empirical evidence.

## Footnotes

1 Government Office for Science, ‘Trend Deck 2021: Urbanisation’ (28 June 2021) <https://www.gov.uk/government/publications/trend-deck-2021-urbanisation/trend-deck-2021-urbanisation> accessed 27 January 2022.

2 ibid.

3 Michael Marmot and others, ‘Closing the Gap in a Generation: Health Equity Through Action on the Social Determinants of Health’ (2008) 372 The Lancet 1661.

4 The purpose of the article is to look at the normative implications of the international human rights framework, rather than questions of justiciability within domestic UK courts. As such, I do not (directly) address or limit my discussions to legal claims being made on the basis of the European Convention on Human Rights under the Human Rights Act 1998. I also refers to rights whose application is not possible within domestic courts because they only apply to the UK in international law (under the International Covenant on Economic and Social Rights and the European Social Charter), such as the right to health and the right to housing.

5 ibid; Michael Marmot, ‘Health Equity in England: The Marmot Review 10 Years On’ (February 2020) <https://www.health.org.uk/publications/reports/the-marmot-review-10-years-on> accessed 27 January 2022.

6 ibid.

7 Cambridge Dictionary <https://dictionary.cambridge.org/fr/dictionnaire/anglais/law> accessed 27 January 2022.

8 John Coggon, *What Makes Health Public?* (CUP 2012).

9 ibid; Marmot (n 3); Marmot (n 5).

10 Marmot (n 3); Marmot (n 5).

11 ibid.

12 ibid.

13 Sridhar Venkatapuram, ‘Global Justice and the Social Determinants of Health’ (2010) 24 Ethics and International Affairs 119.

14 Scott Burris and others, ‘A Transdisciplinary Approach to Public Health Law: The Emerging Practice of Legal Epidemiology’ (2016) 37 Annual Review of Public Health 135.

15 Michel Coleman, ‘Cancer Survival: Global Surveillance Will Stimulate Health Policy and Improve Equity’ (2014) 383 The Lancet 564.

16 Government Office for Science, ‘Trend Deck 2021: Urbanisation’ (28 June 2021) <https://www.gov.uk/government/publications/trend-deck-2021-urbanisation/trend-deck-2021-urbanisation> accessed 1 August 2022.

17 ibid.

18 Scott Burris and others, ‘A Vision of Health Equity in Housing’ (The Legal Levers for Health Equity through Housing Report Series, Center for Public Health Law Research, 2019).

19 Venkatapuram (n 13).

20 Marmot (n 3).

21 World Health Organisation, ‘Noncommunicable Diseases’ (13 April 2021) <https://www.who.int/news-room/fact-sheets/detail/noncommunicable-diseases> accessed 27 January 2022.

22 Adam Sheppard, Nick Croft and Nick Smith, *The Short Guide to Town and Country Planning* (1st ed, Policy Press 2019) 132.

23 Marmot (n 3).

24 Marmot (n 3); Marmot (n 5).

25 Adam Sheppard and others, *The Essential Guide to Planning Law: Decision-Making and Practice in the UK* (1st ed, Policy Press 2017) 119.

26 Ministry of Housing, Communities and Local Government, ‘National Planning Policy Framework’ (NPPF) (published 27 March 2012, updated 20 July 2021) <https://www.gov.uk/government/publications/national-planning-policy-framework–2> accessed 27 January 2022, para 2; Planning and Compulsory Purchase Act 2004 c.5, s 38(6).

27 NPPF ibid para 8(a).

28 House of Commons Library, ‘Planning for the Future: Planning Policy Changes in England in 2020 and Future Reforms’ (11 February 2022) <https://commonslibrary.parliament.uk/research-briefings/cbp-8981/> accessed 1 August 2022.

29 Jean-Jacques Rousseau, *Du Contrat Social, ou Principes du Droit Politique* (1762).

30 Sheppard, Croft and Smith (n 22); Sheppard and others (n 25).

31 Sheppard refers to a ‘network’ of legislation to show the multiple law areas involved in urban planning in Sheppard, Croft and Smith (n 22) 113.

32 ‘Procurement Policy Note – Taking Account of Social Value in the Award of Central Government Contracts’ (September 2020 PPN 06/20) <https://assets.publishing.service.gov.uk/government/uploads/system/uploads/attachment_data/file/921437/PPN-06_20-Taking-Account-of-Social-Value-in-the-Award-of-Central-Government-Contracts.pdf> accessed 31 January 2022.

33 ‘Grenfell Tower Inquiry: Phase 1 Report Overview’ (October 2019) <https://assets.grenfelltowerinquiry.org.uk/GTI%20-%20Phase%201%20report%20Executive%20Summary.pdf> accessed 27 January 2022.

34 Sheppard, Croft and Smith (n 22).

35 NPPF (n 26) paras 7–14.

36 HM Government, *Net Zero Strategy: Build Back Greener* (Crown Copyright 2021).

37 WHO Europe, *Noncommunicable Diseases and Air Pollution: WHO European High-Level Conference on Noncommunicable Diseases* (WHO Regional Office for Europe 2019).

38 Public Health England, ‘Improving Access to Greenspace: A New Review for 2020’ (March 2020) <https://assets.publishing.service.gov.uk/government/uploads/system/uploads/attachment_data/file/904439/Improving_access_to_greenspace_2020_review.pdf> accessed 27 January 2022; ‘UPSTREAM Project Report: Moving Planetary Health Upstream in Urban Development Decision-Making – a Three-Year Pilot Research Project’ <https://urban-health-upstream.info/wp-content/uploads/2018/12/Upstream_Brochure_WEB_single.pdf > accessed 27 January 2022.

39 WHO Europe, *Urban Green Spaces and Health: A Review of Evidence* (WHO Regional Office for Europe 2016).

40 Public Health England (n 38).

41 Anna Le Gouais and others, ‘A Natural Experimental Study of New Walking and Cycling Infrastructure Across the United Kingdom: The Connect2 Programme’ (2021) 20 Journal of Transport and Health 100968.

42 ‘Tackling the Under-Supply of Housing in England’ (House of Commons Library 14 January 2021) <https://researchbriefings.files.parliament.uk/documents/CBP-7671/CBP-7671.pdf> accessed 27 January 2022.

43 Department for Communities and Local Government, *A Decent Home: Definition and Guidance for Implementation June 2006 – Update* (Crown Copyright 2006); World Health Organisation, *Health Principles of Housing* (WHO 1989).

44 UN Habitat, *Accessibility of Housing: A Handbook of Inclusive Affordable Housing Solutions for Persons with Disabilities and Older Persons* (United Nations Human Settlements Programme 2014).

45 Lawrence Gostin and others, ‘The Legal Determinants of Health: Harnessing the Power of Law for Global Health and Sustainable Development’ (2019) 393 The Lancet 1857; John Coggon, ‘Legal, Moral and Political Determinants within the Social Determinants of Health: Approaching Transdisciplinary Challenges through Intradisciplinary Reflection’ (2020) 13 Public Health Ethics 41; Scott Burris and Vivian Lin, ‘Law and Urban Governance for Health in Times of Rapid Change’ (2021) 36(S1) Health Promotion International i4–i12.

46 Sheppard, Croft and Smith (n 22).

47 Jack Donnelly, ‘The Relative Universality of Human Rights’ (2007) 29 Human Rights Quarterly 281.

48 ibid 287–288.

49 UN General Assembly, *Vienna Declaration and Programme of Action*, 12 July 1993, A/CONF.157/23.

50 For instance, UN General Assembly, *International Covenant on Economic, Social and Cultural Rights* (ICESCR), 16 December 1966, United Nations, Treaty Series, vol 993 p 3 (preamble); UN Committee on Economic, Social and Cultural Rights (CESCR), *General Comment No 3: The Nature of States Parties’ Obligations (art 2, para 1, of the Covenant)*, 14 December 1990, E/1991/23; CESCR, *General Comment No 14: The Rights to the Highest Attainable Standard of Health (art 12 of the Covenant)*, 11 August 2000, E/C.12/2000/4.

51 UN Office of the High Commissioner for Human Rights, ‘What Are Human Rights?’ <https://www.ohchr.org/en/issues/pages/whatarehumanrights.aspx#:∼:text=All%20human%20rights%20are%20indivisible,economic%2C%20social%20and%20cultural%20rights.> accessed 27 January 2022.

52 Paul Hunt, ‘Missed Opportunities: Human Rights and the Commission on Social Determinants of Health’ (2009) 16 Global Health Promotion 36.

53 UNFPA, ‘Human Rights Principles’ (2005) <https://www.unfpa.org/fr/node/9206> accessed 27 January 2022.

54 Venkatapuram (n 13); Hunt (n 52); Audrey Chapman, ‘The Social Determinants of Health, Health Equity, and Human Rights’ (2010) 12 Health and Human Rights Journal 17.

55 Venkatapuram (n 13); Chapman ibid.

56 Jonathan Mann and others, ‘Health and Human Rights’ (1994) 1 Health and Human Rights Journal 6, 19.

57 UN General Assembly, *Universal Declaration of Human Rights*, 10 December 1948, 217 A (III), preamble, art 25; ICESCR (n 50) art 12; UN General Assembly, *Convention on the Rights of the Child*, 20 November 1989, United Nations, Treaty Series, 1577, p 3, art 24; UN General Assembly, *Convention on the Elimination of All Forms of Discrimination Against Women*, 18 December 1979, United Nations, Treaty Series, 1249, p 13,  art 12; UN General Assembly, *Convention on the Rights of Persons with Disabilities*, 24 January 2007, A/RES/61/106 art 25.

58
*Council of Europe, European Social Charter* (Revised), 3 May 1996, ETS 163.

59 General Comment 14 (n 50).

60 ibid para 4.

61 Paul Hunt and Gunilla Backman, ‘Health Systems and the Right to the Highest Attainable Standard of Health’ (2008) 10 Health and Human Rights Journal 1.

62 Gostin (n 45).

63 NPPF (n 26) para 8(b).

64 ibid para 2; Planning and Compulsory Purchase Act 2004 (n 26) s 38(6).

65 NPPF ibid paras 7 and 8.

66 ibid para 9.

67 Gostin (n 45).

68 NPPF (n 26) para 8(a); see Tackling the Under-Supply of Housing in England (n 42).

69 Lawrence Gostin, ‘Meeting Basic Survival Needs of the World’s Least Health People: Toward a Framework Convention on Global Health’ (2008) 96 Georgetown Law Journal 331.

70 World Health Organization & Finland. Ministry of Social Affairs and Health, *Health in All Policies: Helsinki Statement* (2014).

71 ibid; Venkatapuram (n 13).

72 For example, access to natural light in all main living areas and bedrooms (principle c), inclusive, accessible, and adaptable places to suit the needs of everyone, including the protected characteristics of the Equality Act (principle d), or the minimisation of harmful impacts of air pollution on human health and the environment (principle j). Healthy Homes HL Bill (session 2021–2022) [133].

73 Gostin (n 45).

74 ibid.

75 Well-being of Future Generations (Wales) Act 2015 (anaw 2).

76 Haydn Davies, ‘The Well-being of Future Generations (Wales) Act 2015—A Step Change in the Legal Protection of the Interests of Future Generations?’ (2017) 29 Journal of Environmental Law 165.

77 Ole Peter Ottersen and others, ‘The Political Origins of Health Inequity: Prospects for Change’ (2014) 383 The Lancet 630.

78 ibid; Gostin (n 45).

79 Gostin (n 45).

80 The third challenge relates to the multiplicity of international agencies in global health governance, Gostin (n 45) 1880.

81 Gostin (n 45) 1877.

82 Department for Communities and Local Government, *Devolution: A Mayor for the West of England. What does it Mean?* (Department for Communities and Local Government 2017) <https://assets.publishing.service.gov.uk/government/uploads/system/uploads/attachment_data/file/608525/Plain_English_Guides_to_Devolution_West_of_England.pdf> accessed on 4 August 2022.

83 Burris and Lin (n 45).

84 Gostin (n 45) 1878.

85 Grenfell report (n 33).

86 UPSTREAM report (n 38).

87 Burris and Lin (n 45).

88 Gostin (n 45).

89 ibid.

90 ibid.

91 World Health Organisation, *Global Action Plan for the Prevention and Control of Noncommunicable Diseases 2013-2020* (World Health Organisation 2013).

92 Gostin (n 45) 1875.

93 ibid.

94 ibid.

95 Marmot (n 3).

96 Gostin (n 45).

97 CESCR, *General Comment No 4: The Right to Adequate Housing (art 11(1) of the Covenant)*, 13 December 1991, E/1992/23; CESCR, *General Comment No 12: The Right to Adequate Food (art 11 of the Covenant)*, 12 May 1999; CESCR, *General Comment No 13: The Right to Education (art 13 of the Covenant)*, 8 December 1999, E/C.12/1999/10; *Preliminary report of the Special Rapporteur on the Right to Education, Katarina Tomasevski, submitted in accordance with Commission on Human Rights resolution 1998/33*, 13 January 1999, E/CN.4/1999/49 para 50; General Comment 14 (n 50).

98 General Comment 4 (n 97) para 8.

99 ibid.

100 ibid para 8(d).

101 World Health Organisation (n 43).

102 General Comment 4 (n 97) para 8(f).

103 NPPF (n 26) paras 88 and 92(a).

104 Courts and Tribunals Judiciary, ‘Ella Kissi-Debrah: Prevention of Future Deaths Report’ (20 April 2021) 2021-0113 <https://www.judiciary.uk/publications/ella-kissi-debrah/> accessed 31 January 2022.

105 For instance, General Comment 14 (n 50); Alicia Ely Yamin, ‘Will We Take Suffering Seriously? Reflections on What Applying a Human Rights Framework to Health Means and Why We Should Care’ (2008) 10 Health and Human Rights 45.

106 ICESCR (n 50) arts 2(2) and 3.

107 Equality Act 2010, c.15.

108 For instance, ICESCR (n 50) art 2(2).

109 Human Rights Act 1998, c.42.

110 Equality Act 2010, c.15, s 19.

111 Human Rights Act 1998, s 6(1).

112 For instance, General Comment 14 (n 50); Jonathan Wolff, *The Human Rights to Health* (Norton Paperback 2012).

113 Human Rights Act 1998, s 8.

114 International Commission of Jurists, Maastricht Guidelines on Violations of Economic, Social and Cultural Rights, 26 January 1997, para 23.

115 Alicia Ely Yamin, ‘Beyond Compassion: The Central Role of Accountability in Applying a Human Rights Framework to Health’ (2008) 10 Health and Human Rights Journal 1.

116 Well-being of Future Generations (n 75).

117 Planning Act 2008, ss 47–49.

118 Alicia Ely Yamin and Andrés Constantin, ‘A Long and Winding Road: The Evolution of Applying Human Rights Frameworks to Health’ (2017) 49 Georgetown Journal of International Law 191.

119 Gita Parihar, ‘An Environment for Change: Using Law to Protect the Planet’ (2014) 13 Journal of Planning and Environment Law 31.

120 Philippine Human Rights Information Center, *Monitoring Economic, Social and Cultural Rights: The Philippine Experience, Phase One 2* (1997) cited in Maria Green, ‘What We Talk About When We Talk About Indicators: Current Approaches to Human Rights Measurement’ (2001) 23 Human Rights Quarterly 1062, 1095.

121 Gillian MacNaughton, ‘Human Rights: Frameworks, Strategies and Tools for the Poverty Lawyer’s Toolbox’ (2011) 44 Clearinghouse Review 437.

122 Chapman (n 54).

123 For instance, General Comment 14 (n 50) paras 18 and 19.

124 Equality Act 2010, c.15, s 19.

125 Marmot (n 3).

126 Chapman (n 54); Marmot (n 3).

127 Chapman (n 54); General Comment 14 (n 50).

128 Marmot (n 3).

129 For instance, the UN treaty-based monitoring system prioritises dialogue between States parties, human rights bodies, and civil society, aiming to improve the implementation of human rights rather than shaming.

130 Donnelly (n 47).

131 Yamin and Constantin (n 118); Chapman (n 54); Art Hendriks and Brigit Toebes, ‘Towards a Universal Definition of the Right to Health’ (1998) 17 Medicine and Law 319, 326.

132 Yamin and Constantin (n 118).

